# Supplementary material for: A novel multi-agent simulation based particle swarm optimization algorithm
Source: PLoS One. 2022 Oct 13;17(10):e0275849. doi: 10.1371/journal.pone.0275849 (PMC9560124; doi:10.1371/journal.pone.0275849)
Supplement: Attachment — Submitted filename: Response to Reviewers.docx [file pone.0275849.s001.docx]

**Response Letter**

Dear Editors and Reviewers

Thank you for your careful review and constructive comments regarding our manuscript. These comments are all valuable and helpful for improving our paper. All the authors have seriously discussed about all the comments. We have tried our best to revise the manuscript in accordance with the comments and highlighted in yellow all the amends in our revised manuscript. The point-by-point replies are listed below. We sincerely appreciate your help.

Reply to Journal Requirements

**Point 1:**

*Please ensure that your manuscript meets PLOS ONE's style requirements, including those for file naming.*

**Reply:**

We have carefully checked the manuscript of the article. Our manuscript meets PLOS ONE's style requirements.

**Point 2:**

*We note that Figure 8 in your submission contain [map/satellite] images which may be copyrighted.*

**Reply:**

We have removed the figure you mentioned. Since we have adjusted the figures in the revised manuscript, the mark numbers have changed a bit. Figure 5 in our revised version also contained [map/satellite] images (of the same areas as the figure you mentioned), which came from USGS EROS (thank you for your recommendation). We have followed the requirements of USGS EROS and included the following acknowledgment statement in the caption of Figure 5:

*Map services and data available from the U.S. Geological Survey.*

Reply to Reviewer 1 Comments

Comment 1

*First off, please clearly state the gap targeted in this paper at the end of introduction and list down the hypotheses.*

**-Response & Revision:**

Thank you very much for the valuable feedback on our Introduction. We have modified the end of our Introduction as follows:

*We can see from the literature that although there are many studies on improving SPSO, there are not so many attempts to combine proposed algorithms with MAS and apply them to practical problems. In fact, utilizing improved algorithms in simulation-optimization systems is necessary because complex tasks require high-performance optimizers, and SPSO may not meet this requirement. Besides, existing studies on the combination of MAS and PSO may vary in engineering fields, yet they all designed the system based on specific problems of their own research field without summarizing a general and universal framework, which is field-independent and can be implemented in various settings. Therefore, this paper focuses on a more in-depth exploration of improving PSO and integrating it into MAS to solve practical problems. The main contributions of this paper are as follows:*

*1. We proposed an improved particle swarm optimization algorithm called CoPSO according to the biological nature of birds. CoPSO has a multi-level structure and a competition mechanism, aiming to improve performance by enhancing exploration while balancing exploitation.*

*2. We proposed a general approach to integrate CoPSO into MAS called MAS-CoPSO. MAS-CoPSO is supposed to achieve a very close combination between simulation and optimization, and the application should not be limited to certain engineering fields and specific problems.*

*3. We conducted a series of comparison experiments to substantiate the validity of CoPSO.*

*4. We implemented MAS-CoPSO in a case study of the Sanchi oil spill accident to solve the response planning problem.*

Comment 2

*In terms of research method and design, there is not much in the paper.*

**-Response & Revision:**

We appreciate a lot for your comments on the formation of the paper. To address this problem, we have adjusted the structure of the paper by adding section *Methods and materials* after *Introduction*. There are three subsections in *Methods and materials*, namely, *Multi-agent Simulation*, *The proposed CoPSO algorithm*, and *The MAS-CoPSO approach*. These three parts have described the research method and design of the paper in detail.

Comment 3

*The comparative algorithms in the experiments are not properly acknowledged and cited.*

**-Response & Revision:**

Thank you for pointing out this problem. We have four comparative algorithms, namely, the standard formation of PSO (SPSO) [1], the random weight method (RPSO) [2], the constriction factor approach (CPSO) [3], and the incremental particle swarm optimizer (IPSO) [4]. We briefly mentioned these algorithms in *Introduction* without proper acknowledgments and citations in the experiments. For modification, we have cited these algorithms and described their contents in section *Experiments and analysis of CoPSO*.

Comment 4

*I also suggest adding some figures to better articular the content as the paper looks very dry at the moment.*

**-Response & Revision:**

Thank you for your constructive suggestion. Firstly, we have added Fig 1 and Fig 2 (the mark numbers subject to the revised manuscript) for a better description of CoPSO. They are both two-dimensional schematic diagrams. Fig 1 illustrates how the particles’ levels change during the iterations. Fig 2 depicts how the competition mechanism functions in the algorithm. We have also added Fig 5 in *Case study of MAS-CoPSO* to enrich the background description. Fig 5 is a geographical map illustrating the approximate location where the Sanchi oil spill accident happened. In addition, we have modified some original figures. Fig 4 in the revised manuscript contains subgraphs of benchmark functions’ converging behaviors and their swarm diversity variations. We have also redrawn Fig 7 to depict the simulation process of the case study more clearly. Fig 8 and Fig 9 are modified for better comparison.

Comment 5

*Analysis of the results is missing in the paper. There is a big gap between the results and conclusion. There should be the result analysis between these two sections. After comparing the numerical methods, you have to be able to analyze the results and relate them to their structures. It would be interesting to have your thoughts on why the method works that way? Such analyses would be the core of your work where you prove your understanding of the reason behind the results. You can also link the findings to the hypotheses of the paper. Long story short, this paper requires a very deep analysis from different perspectives.*

**-Response & Revision:**

Thank you for pointing out this problem. As we have adjusted the formation of the paper, modifications for this comment can be found in section *Experiments and analysis of CoPSO*. Considering some exceptional results may hugely affect the “Mean” performance and conceal the algorithms’ properties, we have added the comparison item “Median” in Table 2. Further, we have also introduced the concept of swarm diversity [5] to analyze the algorithms’ exploration abilities. After comparing the diversity evolution and the convergence behavior of each algorithm on all the benchmarks in Fig 4, we have discussed the results based on the architectures of the algorithms and concluded that the multi-level structure and the competition mechanism did improve the performance of CoPSO by enhancing exploration while balancing exploitation. A detailed analysis can be found in *Results and discussion* of *Comparison experiments*.

Comment 6

*There is no statistical test to judge about the significance of the numerical method’s results. Without such a statistical test, the conclusion cannot be supported.*

**-Response & Revision:**

Thank you for this feedback. For modification, as recommended by Yang et al. [5], we have conducted Wilcoxon rank sum test for the statistical analysis between CoPSO and the other algorithms with a significance level of 0.05. The p-values were listed in Table 2. Results of the statistical test have supported our conclusion. A detailed discussion can be found in *Results and discussion* of *Comparison experiments*.

Comment 7

*There is no discussion on the cost effectiveness of the proposed method. What is the computational complexity? What is the runtime? Please include such discussions. You can also use the big oh notation to show the computation complexity.*

**-Response & Revision:**

Thank you for this comment. We have added subsection *Computational complexity analysis* in *Experiments and analysis of CoPSO* to make up for this part. According to Yang et al. [5], given a fixed number of fitness evaluations, the computational complexity of an evolutionary algorithm is generally calculated by analyzing the extra cost in each generation without considering the cost of function evaluations, which is problem-dependent. As CoPSO inherits the simple structure of SPSO, we have analyzed the computational complexity (in terms of time and space, using the big oh notation) of CoPSO by comparing it with SPSO. We have also compared the runtime of all the algorithms and analyzed the results listed in Table 3.

Comment 8

*Some mathematical notations and Lemma presentations are not rigorous enough to correctly understand the contents of the paper. The authors are requested to recheck all the definition of variables and further clarify these equations.*

**-Response & Revision:**

Thank you for pointing out this problem. We have rechecked the paper and modified the following ambiguous mathematical notations and lemma presentations (the mark numbers of the tables and equations subject to the revised manuscript):

1. As the position and velocity of a particle (for example, particle $i$) are supposed to be vectors, we have modified the presentation of these two variables to the bold form $\boldsymbol{x}_{\boldsymbol{i}}=(x_{i}^{1}, x_{i}^{2}, \ldots, x_{i}^{D})$ and $\boldsymbol{v}_{\boldsymbol{i}}=(v_{i}^{1}, v_{i}^{2}, \ldots, v_{i}^{D})$, where the superscripts indicate dimensions. We have also modified the personal best position of particle $i$ and the global best position to $\boldsymbol{pbest}_{\boldsymbol{i}}=({pbest}_{i}^{1}, {pbest}_{i}^{2}, \ldots, {pbest}_{i}^{D})$ and $\boldsymbol{gbest}=({gbest}^{1}, {gbest}^{2}, \ldots, {gbest}^{D})$, respectively. For a more concise expression, we have modified the denotation of the level of particle $i$ to $l_{i}$ with an upper bound of $L_{max}$. Finally, we have further clarified Eq (1), Eq (2), and Algorithm 1.
2. We have added the definitions of $n$ (the problem dimension) and $f_{min}$ (the global optimum) in the note of Table 1.
3. We have added the units of $\rho_{\omega}$ and $\rho_{0}$ in Eq (3).
4. We have supplemented the definitions of $A$, $t$ and $V$ in Eq (4).
5. We have supplemented the definitions of $F_{E}$ and added the calculation of $T_{O}$ and $T_{G}$ in Eq (5).
6. We have added the unit of $\mu_{0}$ and the explanation of $AC$ in Eq (6), as well as the unit of $\mu$ and the calculation of $Y_{W}$ in Eq (7).
7. We have supplemented the definitions of $V_{D}$ and added the unit of $\zeta_{t}$ in Eq (8). We have also modified the presentation of Eq (8) to avoid the misunderstanding of variable $A$ and variable $V$.
8. We have supplemented the definitions of ${ORR}_{i}$ in Eq (9) and ${ST}_{k}$ in Eq (10). We have also added the explanation of parameter $\alpha$ and $\beta$ in Eq (9), as well as the calculation of $A_{k}$ in Eq (10).
9. We have supplemented the definitions and the calculations of $V_{k}$, $V_{k0}$, ${EV}_{k}$, $DV_{k}$, and ${SV}_{i}$ in Eq (11).
10. We have added the definitions of $V_{step}$ in Eq (12), ${F_{E}}_{k}$ in Eq (14), and $\mu_{k}$ in Eq (16). We have also modified Eq (13), Eq (14), and Eq (16) for better presentation.
11. We have stated our objective in text form for a better description of Eqs (12)~(18) (*The aim of our system is to clean up the polluted sea area as soon as possible. Therefore, based on our method, given a fixed simulation time step, we will maximize the oil volume loss during each step, which is composed of the evaporation loss, the dispersion loss and the recovered volume.*).
12. We have added the meaning of each result item in the note of Table 7.

**References in replies:**

[1] Kennedy J, Eberhart R. Particle swarm optimization. In: Proceedings of ICNN’95-international conference on neural networks. vol. 4. IEEE; 1995. p. 1942–1948.

[2] Eberhart RC, Shi Y. Tracking and optimizing dynamic systems with particle swarms. In: Proceedings of the 2001 congress on evolutionary computation (IEEE Cat. No. 01TH8546). vol. 1. IEEE; 2001. p. 94–100.

[3] Clerc M, Kennedy J. The particle swarm-explosion, stability, and convergence in a multidimensional complex space. IEEE transactions on Evolutionary Computation. 2002;6(1):58–73.

[4] De Oca MAM, Stutzle T, Van den Enden K, Dorigo M. Incremental social learning in particle swarms. IEEE Transactions on Systems, Man, and Cybernetics, Part B (Cybernetics). 2010;41(2):368–384.

[5] Yang Q, Chen WN, Da Deng J, Li Y, Gu T, Zhang J. A level-based learning swarm optimizer for large-scale optimization. IEEE Transactions on Evolutionary Computation. 2017;22(4):578–594.

Reply to Reviewer 2 Comments

Comment 1

*Please clear what are the main research gaps in Abstract.*

**-Response & Revision:**

Thank you very much for pointing out the problems in our *Abstract*. For modification, we have stated the main research gaps and the contributions of our paper as follows:

*Recently, there have been considerable researches on combining multi-agent simulation and particle swarm optimization in practice. However, most existing studies are limited to specific engineering fields or problems without summarizing a general and universal combination framework. Moreover, particle swarm optimization can be less effective in complex problems due to its weakness in balancing exploration and exploitation. Yet, it is not common to integrate MAS with improved versions of the algorithm. Therefore, this paper proposed an improved particle swarm optimization algorithm called CoPSO, introducing a multi-level structure and a competition mechanism to enhance exploration while balancing exploitation. Further, we integrated CoPSO into MAS by a problem-independent approach called MAS-CoPSO, aiming to simulate realistic scenarios dynamically and solve related optimization problems simultaneously.*

Comment 2

*The CoPSO algorithm is not clear and can be described more.*

**-Response & Revision:**

Thank you for this feedback. We have modified subsection *Improved particle swarm optimization algorithm with competition mechanism* to address this problem, a detailed description can be found there. Firstly, we have introduced the standard formation of PSO and stated our motivation, i.e., improving the performance of PSO by enhancing exploration while balancing exploitation. Inspired by the biological nature of birds, we have proposed a multi-level structure and a competition mechanism in CoPSO. We divide particles into different levels and stipulate that the closer a particle is to a possible global optimum, the higher its level. If a particle doesn't update its personal best position in $\mu$ continuous iterations, it will upgrade. Particles of different levels learn at different acceleration constants. Fig 1 is a two-dimensional schematic diagram of the variations of particles’ levels during the iterations.

However, a higher level doesn’t always a mean closer distance to the global optimum, it can also indicate that the particle has fallen into local optima. The basic idea of the competition mechanism is to replace particles of extremely high levels with randomly generated new ones. Thus, the algorithm gets the potential to escape from local optima and explore the unknown search space. Fig 2 illustrates how the competition mechanism functions, particles in Fig 2a are trapped in local areas, once their levels get too high, we will regenerate new particles to replace them. From Fig 2b we can see that these new particles have located the right promising area.

The pseudo-code of CoPSO is shown in Algorithm 1.

Comment 3

*The major novelties of this work should be listed in the introduction.*

**-Response & Revision:**

Thank you for pointing out this problem. We have listed the novelties of the paper in the end of our Introduction as follows:

*The main contributions of this paper are as follows:*

*1. We proposed an improved particle swarm optimization algorithm called CoPSO according to the biological nature of birds. CoPSO has a multi-level structure and a competition mechanism, aiming to improve performance by enhancing exploration while balancing exploitation.*

*2. We proposed a general approach to integrate CoPSO into MAS called MAS-CoPSO. MAS-CoPSO is supposed to achieve a very close combination between simulation and optimization, and the application should not be limited to certain engineering fields and specific problems.*

*3. We conducted a series of comparison experiments to substantiate the validity of CoPSO.*

*4. We implemented MAS-CoPSO in a case study of the Sanchi oil spill accident to solve the response planning problem.*

Comment 4

*Next section after introduction should be methods and materials.*

**-Response & Revision:**

Thank you for your constructive comments on the formation of the paper. We have added section *Methods and materials* after *Introduction*. There are three subsections in *Methods and materials*, namely, *Multi-agent Simulation*, *The proposed CoPSO algorithm*, and *The MAS-CoPSO approach*. These three parts have described the research method and design of the paper in detail.

Comment 5

*In the section of case study, please describe the technical details of benchmarks only not methods details.*

**-Response & Revision:**

Thank you for the valuable feedback. Based on the modeling of an oil spill accident, we conducted MAS-CoPSO for response planning and used the shortest distance selection approach (SDS) as a contrast. SDS is commonly used in real oil spill response emergencies [1], which makes the comparison reasonable. We have added a more detailed description of the procedure of SDS in subsection *The performance of the MAS-CoPSO-based system*. Instead of calling the CoPSO module, SDS used the distances between ships and oil slicks as the measure for planning, which was the main difference between these two approaches. When initializing the simulation process, we assigned each response team with the closest oil slick to clean. Before each time step, we would check whether the slick was cleaned up, if the oil volume was below the set threshold, the ship would head to the closest one in the remaining slicks, otherwise, it would stay at the current location until completing the recovery.

Comment 6

*It can be helpful to have a better understanding of CoPSO with adding some relevant references and adaptive PSO as well such as a) FAIPSO: fuzzy adaptive informed particle swarm optimization. Neural Computing and Applications. 2013 Dec;23(1):95-116. b) "A new kind of PSO: predator particle swarm optimization." International Journal on Smart Sensing and Intelligent Systems 5, no. 2 c) An adaptive particle swarm optimizer with decoupled exploration and exploitation for large scale optimization." Swarm and Evolutionary Computation 60 (2021): 100789.*

**-Response & Revision:**

Thank you for providing these papers. We have cited it in our *Introduction*.

**References in replies:**

[1] Ye X, Chen B, Li P, Jing L, Zeng G. A simulation-based multi-agent particle swarm optimization approach for supporting dynamic decision making in marine oil spill responses. Ocean & Coastal Management. 2019;172:128–136.

[2] Neshat M. FAIPSO: fuzzy adaptive informed particle swarm optimization. Neural Computing and Applications. 2013;23(1):95–116.

[3] Neshat M, Sargolzaei M, Masoumi A, Najaran A. A new kind of PSO: predator particle swarm optimization. International Journal on Smart Sensing and Intelligent Systems. 2012;5(2).

[4] Li D, Guo W, Lerch A, Li Y, Wang L, Wu Q. An adaptive particle swarm optimizer with decoupled exploration and exploitation for large scale optimization. Swarm and Evolutionary Computation. 2021;60:100789.
