# Supplementary material for: A novel multi-agent simulation based particle swarm optimization algorithm
Source: PLoS One. 2022 Oct 13;17(10):e0275849. doi: 10.1371/journal.pone.0275849 (PMC9560124; doi:10.1371/journal.pone.0275849)
Supplement: Attachment — Submitted filename: Response to Reviewers.docx [file pone.0275849.s002.docx]

**Response Letter**

Dear Editors and Reviewers

Thank you for your careful review and constructive comments regarding our manuscript. These comments are all valuable and helpful for improving our paper. All the authors have seriously discussed about all the comments. We have tried our best to revise the manuscript in accordance with the comments and highlighted in yellow all the amends in our revised manuscript. The point-by-point replies are listed below. We sincerely appreciate your help.

Reply to Journal Requirements

*Please review your reference list to ensure that it is complete and correct. If you have cited papers that have been retracted, please include the rationale for doing so in the manuscript text, or remove these references and replace them with relevant current references. Any changes to the reference list should be mentioned in the rebuttal letter that accompanies your revised manuscript. If you need to cite a retracted article, indicate the article’s retracted status in the References list and also include a citation and full reference for the retraction notice.*

**Reply:**

We have removed some references according to the reviewer’s comment (i.e., references 21, 23, 25, 26, and 27 of the original manuscript). We have carefully checked the revised version and the reference list is complete and correct. None of the remaining references have been retracted.

Reply to Reviewer 1 Comments

Comment 1

*The results of your comparative study should be discussed in-depth and with more insightful comments on the behavior of your algorithm on various case studies. Discussing results should not mean reading out the tables and figures once again.*

**-Response & Revision:**

Thank you very much for the valuable feedback. We have modified the discussion of the comparative study results as follows.

Comment 2

*Avoid lumping references as in [x, y] and all other. Instead summarize the main contribution of each referenced paper in a separate sentence. For scientific and research papers, it is not necessary to give several references that say exactly the same. Anyway, that would be strange, since then what is innovative scientific contribution of referenced papers? For each thesis state only one reference.*

**-Response & Revision:**

Thank you for pointing out this problem. We have checked the references and removed items containing the same information as those kept in the revised manuscript (i.e., references 21, 23, 25, 26, and 27 of the original version).

Comment 3

*Avoid using first person.*

**-Response & Revision:**

Thank you for pointing out this problem. We have carefully checked the whole manuscript and modified related content.

Comment 4

*Avoid using abbreviations and acronyms in title, abstract, headings and highlights.*

**-Response & Revision:**

Thank you for your constructive suggestion. We have carefully checked these parts and modified related expressions to avoid using abbreviations and acronyms.

Comment 5

*Please avoid having heading after heading with nothing in between, either merge your headings or provide a small paragraph in between.*

**-Response & Revision:**

Thank you for pointing out this problem. We have noticed that this problem exists at the beginning of some sections and subsections. For modification, we have provided brief summaries in between.

Comment 6

*The first time you use an acronym in the text, please write the full name and the acronym in parenthesis. Do not use acronyms in the title, abstract, chapter headings and highlights.*

**-Response & Revision:**

Thank you for this feedback. We have carefully checked the manuscript and modified related content.

Comment 7

*The results should be further elaborated to show how they could be used for the real applications.***-Response & Revision:**

Thank you for this comment. Our method has the potential to be used for real applications mainly because: 1) case study results indicate its superiority over SDS (which is commonly used in real situations); 2) it can still function in more complex scenarios; 3) it can be easily extended according to the users’ requirements and achieve a comprehensive simulation of reality. We have further discussed the results as follows.
