# Supplementary material for: A novel multi-agent simulation based particle swarm optimization algorithm
Source: PLoS One. 2022 Oct 13;17(10):e0275849. doi: 10.1371/journal.pone.0275849 (PMC9560124; doi:10.1371/journal.pone.0275849)
Supplement: Attachment — Submitted filename: Response to Reviewers.docx [file pone.0275849.s003.docx]

**Response Letter**

Dear Editors and Reviewers

Thank you for your careful review and constructive comments regarding our manuscript. These comments are all valuable and helpful for improving our paper. All the authors have seriously discussed about all the comments. We have tried our best to revise the manuscript in accordance with the comments. The point-by-point replies are listed below. We sincerely appreciate your help.

Reply to Journal Requirements

*Please review your reference list to ensure that it is complete and correct. If you have cited papers that have been retracted, please include the rationale for doing so in the manuscript text, or remove these references and replace them with relevant current references. Any changes to the reference list should be mentioned in the rebuttal letter that accompanies your revised manuscript. If you need to cite a retracted article, indicate the article’s retracted status in the References list and also include a citation and full reference for the retraction notice.*

**Reply:**

We have not made any changes to the reference list and the list is complete and correct. None of the cited papers have been retracted.

Reply to Reviewer 1 Comments

Comment 1

*Are all the images used in this work copyrights free? If not, have the authors obtained proper copyrights permission to re-use them? Please kindly clarify, and this is just to ensure all the figures are fine to be published in this work.*

**-Response & Revision:**

Thank you very much for the comment. We have carefully checked the images used in this work. All the images are copyrights free.

Comment 2

*Also, the list of references should be carefully checked to ensure consistency with between all references and their compliances with the journal policy on referencing.*

**-Response & Revision:**

Thank you for the suggestion. We have checked the reference list according to the manuscript content and the journal policy. The reference list is complete and correct. None of the cited papers have been retracted.
